# Benzoic acid–3,4-bis­[(pyridin-3-ylmeth­yl)amino]­cyclo­but-3-ene-1,2-dione (1/2)

**DOI:** 10.1107/S1600536812000220

**Published:** 2012-01-14

**Authors:** Andreas Lemmerer, Susan A. Bourne

**Affiliations:** a Molecular Sciences Institute, School of Chemistry, University of the Witwatersrand, Johannesburg, PO Wits 2050, South Africa; b Centre for Supramolecular Chemistry Research, Department of Chemistry, University of Cape Town, Rondebosch 7701, South Africa

## Abstract

In the title co-crystal, C_16_H_14_N_4_O_2_·2C_7_H_6_O_2_, the 3,4-bis­[(pyridin-3-ylmeth­yl)amino]­cyclo­but-3-ene-1,2-dione squareamide mol­ecules assemble into chains along the *b* axis *via* N—H⋯O hydrogen bonds. The benzoic acid mol­ecules then hydrogen bond to the pyridine rings *via* O—H⋯N hydrogen bonds, supported by weaker C—H⋯O hydrogen bonds, forming extended ribbons. The asymmetric unit consists of a half squareamide mol­ecule, sitting on a special position around a twofold axis, and one benzoic acid mol­ecule on a general position.

## Related literature

For the synthesis of related squareamides and co-crystals, see: Liu *et al.* (2002 ▶).
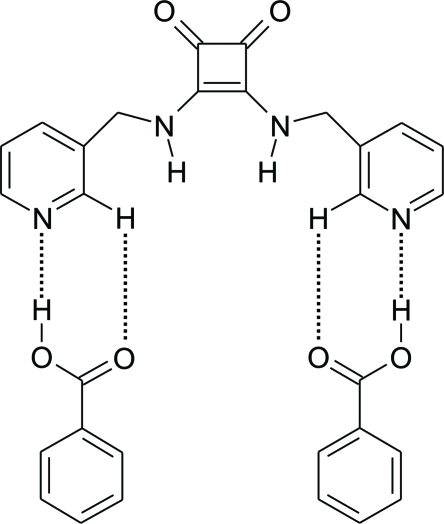


## Experimental

### 

#### Crystal data

C_16_H_14_N_4_O_2_·2C_7_H_6_O_2_
*M* *_r_* = 538.55
Monoclinic, 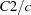

*a* = 24.617 (5) Å
*b* = 6.0285 (12) Å
*c* = 17.806 (4) Å
β = 93.08 (3)°
*V* = 2638.6 (9) Å^3^
*Z* = 4
Mo *K*α radiation
μ = 0.10 mm^−1^
*T* = 173 K
0.49 × 0.16 × 0.14 mm

#### Data collection

Nonius KappaCCD area-detector diffractometer
Absorption correction: integration (*XPREP*; Bruker, 2004 ▶) *T*_min_ = 0.944, *T*_max_ = 0.989
15016 measured reflections
3166 independent reflections
2110 reflections with *I* > 2σ(*I*)
*R*_int_ = 0.076

#### Refinement

*R*[*F* ^2^ > 2σ(*F* ^2^)] = 0.044
*wR*(*F* ^2^) = 0.116
*S* = 0.99
3166 reflections
190 parameters
H atoms treated by a mixture of independent and constrained refinement
Δρ_max_ = 0.23 e Å^−3^
Δρ_min_ = −0.24 e Å^−3^

### 

Data collection: *COLLECT* (Nonius, 2000 ▶); cell refinement: *DENZO-SMN* (Otwinowski & Minor, 1997 ▶); data reduction: *DENZO-SMN*; program(s) used to solve structure: *SHELXS97* (Sheldrick, 2008 ▶); program(s) used to refine structure: *SHELXL97* (Sheldrick, 2008 ▶); molecular graphics: *ORTEP-3 for Windows* (Farrugia, 1997 ▶) and *DIAMOND* (Brandenburg, 1999 ▶); software used to prepare material for publication: *WinGX* (Farrugia, 1999 ▶) and *PLATON* (Spek, 2009 ▶).

## Supplementary Material

Crystal structure: contains datablock(s) global, I. DOI: 10.1107/S1600536812000220/fy2039sup1.cif

Structure factors: contains datablock(s) I. DOI: 10.1107/S1600536812000220/fy2039Isup2.hkl

Supplementary material file. DOI: 10.1107/S1600536812000220/fy2039Isup3.mol

Additional supplementary materials:  crystallographic information; 3D view; checkCIF report

## Figures and Tables

**Table 1:** Hydrogen-bond geometry (Å, °)

*D*—H⋯*A*	*D*—H	H⋯*A*	*D*⋯*A*	*D*—H⋯*A*
N1—H1⋯O1^i^	0.915 (16)	1.889 (17)	2.7697 (15)	161.0 (13)
O2—H2⋯N2	1.07 (2)	1.57 (2)	2.6380 (15)	178.0 (17)
C2—H2*A*⋯O3	0.95	2.68	3.3341 (18)	127

Symmetry code: (i) 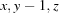.

**Fig. 1.**
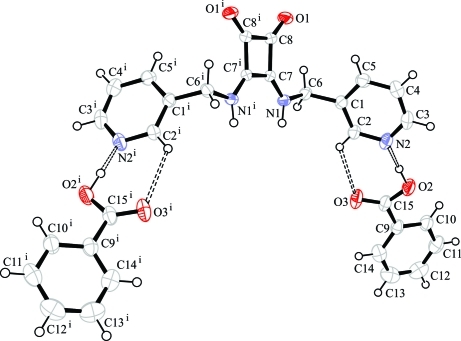
The asymmetric unit of (I) showing the atom numbering scheme. Displacement ellipsoids are shown at the 50% probability level. Atoms with superscript i are generated by the symmetry operation (-x, y, -z + 1/2).

**Fig. 2.**
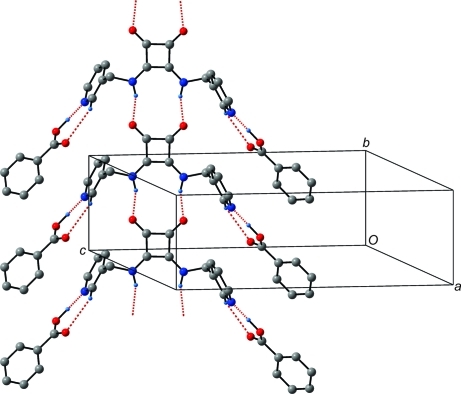
Hydrogen bonding diagram of the ribbons of (I). Intermolecular N—H···O and O—H···N hydrogen bonds are shown as dashed red lines.

## supplementary crystallographic information

### Comment

The title compound is a further example of co-crystals formed by squareamide molecules, in this case 3,4-bis[(pyridin-3-ylmethyl)amino]cyclobut-3-ene-1,2-dione, with carboxylic acid containing co-former molecules. Related co-crystals where the squaureamide molecule has the pyridine N atom in the 4 position are reported by Liu *et al.* (2002). The asymmetric unit consists of one half squareamide molecule, sitting around a twofold axis, and one complete benzoic acid molecule, on a general position (Fig. 1). The squareamide self-assembles into chains using the two N—H···O hydrogen bonds formed from the two amine N—-H groups to the diketones. The pyridine rings then act as hydrogen bond acceptors to the carboxylic acid functional group of the two benzoic acid molecules (Fig. 2).

### Experimental

The title squareamide compound was synthesized according to literature procedures (Liu *et al.*, 2002) by double condensation of diethyl squarate with 1-(pyridin-3-yl)methanamine in ethanol by stirring for 12 h. The resulting solid was filtered and dried. The squareamide was then dissolved in a 1:2 stoichiometric ratio with benzoic acid in a 1/1 *v*/*v* mixture of methanol and water. Plate-like, colourless crystals were harvested after a few days by slow evaporation at ambient conditions.

### Refinement

The C-bound H atoms were geometrically placed with C—H bond lengths of 0.95 Å (aromatic CH) and 0.99 Å (methylene CH_2_) and were refined as riding with *U*_iso_(H) = 1.2*U*_eq_(C). The N-bound and O-bound H atoms were located in the difference map and their coordinates and isotropic displacement parameters were refined freely.

### Crystal data

**Table:**

C_16_H_14_N_4_O_2_·2C_7_H_6_O_2_	*F*(000) = 1128
*M**_r_* = 538.55	*D*_x_ = 1.356 Mg m^−^^3^
Monoclinic, *C*2/*c*	Mo *K*α radiation, λ = 0.71073 Å
Hall symbol: -C 2yc	Cell parameters from 16004 reflections
*a* = 24.617 (5) Å	θ = 0.2–28.3°
*b* = 6.0285 (12) Å	µ = 0.10 mm^−^^1^
*c* = 17.806 (4) Å	*T* = 173 K
β = 93.08 (3)°	Plate, colourless
*V* = 2638.6 (9) Å^3^	0.49 × 0.16 × 0.14 mm
*Z* = 4	

### Data collection

**Table:**

Nonius KappaCCD area-detector diffractometer	2110 reflections with *I* > 2σ(*I*)
2.0° ω scans	*R*_int_ = 0.076
Absorption correction: integration (*XPREP*; Bruker, 2004)	θ_max_ = 28.0°, θ_min_ = 2.3°
*T*_min_ = 0.944, *T*_max_ = 0.989	*h* = −32→26
15016 measured reflections	*k* = −7→7
3166 independent reflections	*l* = −23→21

### Refinement

**Table:**

Refinement on *F*^2^	H atoms treated by a mixture of independent and constrained refinement
Least-squares matrix: full	*w* = 1/[σ^2^(*F*_o_^2^) + (0.0659*P*)^2^ + ] where *P* = (*F*_o_^2^ + 2*F*_c_^2^)/3
*R*[*F*^2^ > 2σ(*F*^2^)] = 0.044	(Δ/σ)_max_ < 0.001
*wR*(*F*^2^) = 0.116	Δρ_max_ = 0.23 e Å^−^^3^
*S* = 0.99	Δρ_min_ = −0.24 e Å^−^^3^
3166 reflections	Extinction correction: *SHELXL97* (Sheldrick, 2008), Fc^*^=kFc[1+0.001xFc^2^λ^3^/sin(2θ)]^-1/4^
190 parameters	Extinction coefficient: 0.0097 (11)
0 restraints	

### Special details

**Table:**

Experimental. Numerical integration absorption corrections based on indexed crystal faces were applied using the *XPREP* routine (Bruker, 2004).
Geometry. All e.s.d.'s (except the e.s.d. in the dihedral angle between two l.s. planes) are estimated using the full covariance matrix. The cell e.s.d.'s are taken into account individually in the estimation of e.s.d.'s in distances, angles and torsion angles; correlations between e.s.d.'s in cell parameters are only used when they are defined by crystal symmetry. An approximate (isotropic) treatment of cell e.s.d.'s is used for estimating e.s.d.'s involving l.s. planes.

### Fractional atomic coordinates and isotropic or equivalent isotropic displacement parameters (Å^2^)

**Table:**

	*x*	*y*	*z*	*U*_iso_*/*U*_eq_	
C1	0.09548 (5)	0.7538 (2)	0.57965 (7)	0.0286 (3)	
C2	0.10973 (5)	0.5561 (2)	0.54679 (7)	0.0333 (3)	
H2A	0.0814	0.4569	0.5308	0.04*	
C3	0.20066 (6)	0.6353 (3)	0.55968 (8)	0.0417 (4)	
H3	0.2373	0.5946	0.5526	0.05*	
C4	0.19060 (6)	0.8346 (3)	0.59354 (8)	0.0430 (4)	
H4	0.2198	0.929	0.61	0.052*	
C5	0.13726 (6)	0.8956 (2)	0.60321 (7)	0.0366 (3)	
H5	0.1293	1.0337	0.6259	0.044*	
C6	0.03667 (5)	0.8124 (2)	0.58837 (7)	0.0307 (3)	
H6A	0.0312	0.9723	0.5777	0.037*	
H6B	0.0136	0.7275	0.5513	0.037*	
C7	0.00897 (5)	0.91990 (19)	0.71280 (7)	0.0261 (3)	
C8	0.01001 (5)	1.16213 (19)	0.71131 (7)	0.0293 (3)	
N1	0.02002 (4)	0.76350 (17)	0.66414 (6)	0.0298 (3)	
H1	0.0195 (6)	0.616 (3)	0.6757 (8)	0.041 (4)*	
N2	0.16108 (5)	0.49629 (19)	0.53623 (6)	0.0384 (3)	
O1	0.02247 (4)	1.30418 (13)	0.66529 (5)	0.0373 (3)	
C9	0.14385 (5)	−0.1060 (2)	0.36020 (7)	0.0350 (3)	
C10	0.18500 (6)	−0.0836 (2)	0.31016 (8)	0.0423 (4)	
H10	0.208	0.0431	0.3128	0.051*	
C11	0.19255 (7)	−0.2448 (3)	0.25655 (9)	0.0513 (4)	
H11	0.2201	−0.2273	0.2217	0.062*	
C12	0.16006 (8)	−0.4312 (3)	0.25359 (9)	0.0573 (5)	
H12	0.1657	−0.543	0.2172	0.069*	
C13	0.11934 (8)	−0.4557 (3)	0.30324 (10)	0.0559 (5)	
H13	0.0972	−0.5848	0.3013	0.067*	
C14	0.11075 (6)	−0.2923 (3)	0.35590 (8)	0.0452 (4)	
H14	0.0821	−0.3078	0.3892	0.054*	
C15	0.13450 (6)	0.0665 (2)	0.41783 (8)	0.0374 (4)	
O2	0.17883 (4)	0.18069 (17)	0.43789 (6)	0.0443 (3)	
H2	0.1708 (7)	0.309 (3)	0.4774 (11)	0.080 (6)*	
O3	0.09068 (4)	0.09691 (18)	0.44435 (6)	0.0484 (3)	

### Atomic displacement parameters (Å^2^)

**Table:**

	*U*^11^	*U*^22^	*U*^33^	*U*^12^	*U*^13^	*U*^23^
C1	0.0315 (7)	0.0303 (7)	0.0247 (6)	0.0022 (5)	0.0069 (5)	0.0042 (5)
C2	0.0308 (7)	0.0342 (7)	0.0355 (7)	0.0025 (6)	0.0083 (6)	−0.0004 (5)
C3	0.0282 (8)	0.0576 (9)	0.0400 (8)	0.0050 (7)	0.0092 (6)	0.0049 (7)
C4	0.0339 (8)	0.0501 (9)	0.0452 (8)	−0.0078 (7)	0.0033 (6)	0.0024 (7)
C5	0.0401 (8)	0.0324 (7)	0.0376 (8)	−0.0007 (6)	0.0065 (6)	0.0003 (6)
C6	0.0320 (7)	0.0289 (7)	0.0319 (7)	0.0048 (5)	0.0081 (5)	0.0008 (5)
C7	0.0227 (6)	0.0211 (6)	0.0353 (7)	0.0006 (5)	0.0073 (5)	0.0000 (5)
C8	0.0282 (7)	0.0219 (6)	0.0384 (7)	0.0012 (5)	0.0084 (5)	0.0004 (5)
N1	0.0355 (6)	0.0203 (6)	0.0348 (6)	0.0017 (5)	0.0130 (5)	0.0013 (4)
N2	0.0335 (7)	0.0443 (7)	0.0384 (7)	0.0092 (5)	0.0103 (5)	−0.0012 (5)
O1	0.0468 (6)	0.0225 (5)	0.0440 (5)	−0.0014 (4)	0.0159 (4)	0.0046 (4)
C9	0.0308 (7)	0.0419 (8)	0.0324 (7)	0.0077 (6)	0.0018 (6)	0.0064 (6)
C10	0.0372 (8)	0.0459 (8)	0.0445 (9)	0.0044 (6)	0.0093 (7)	0.0008 (6)
C11	0.0507 (10)	0.0624 (11)	0.0417 (9)	0.0148 (8)	0.0099 (7)	−0.0028 (8)
C12	0.0702 (12)	0.0560 (11)	0.0441 (9)	0.0146 (9)	−0.0110 (9)	−0.0100 (7)
C13	0.0640 (12)	0.0507 (10)	0.0510 (10)	−0.0084 (8)	−0.0155 (9)	−0.0003 (8)
C14	0.0393 (9)	0.0593 (10)	0.0367 (8)	−0.0032 (7)	−0.0025 (6)	0.0077 (7)
C15	0.0310 (8)	0.0460 (9)	0.0356 (8)	0.0100 (6)	0.0066 (6)	0.0074 (6)
O2	0.0331 (6)	0.0490 (6)	0.0518 (6)	0.0063 (5)	0.0112 (5)	−0.0115 (5)
O3	0.0322 (6)	0.0669 (7)	0.0472 (6)	0.0120 (5)	0.0133 (5)	0.0027 (5)

### Geometric parameters (Å, °)

**Table:**

C1—C2	1.3816 (18)	C8—C8^i^	1.488 (3)
C1—C5	1.3849 (19)	N1—H1	0.915 (16)
C1—C6	1.5061 (18)	C9—C14	1.387 (2)
C2—N2	1.3374 (17)	C9—C10	1.391 (2)
C2—H2A	0.95	C9—C15	1.487 (2)
C3—N2	1.3351 (19)	C10—C11	1.382 (2)
C3—C4	1.373 (2)	C10—H10	0.95
C3—H3	0.95	C11—C12	1.378 (2)
C4—C5	1.383 (2)	C11—H11	0.95
C4—H4	0.95	C12—C13	1.380 (3)
C5—H5	0.95	C12—H12	0.95
C6—N1	1.4612 (16)	C13—C14	1.384 (2)
C6—H6A	0.99	C13—H13	0.95
C6—H6B	0.99	C14—H14	0.95
C7—N1	1.3187 (16)	C15—O3	1.2146 (16)
C7—C7^i^	1.419 (2)	C15—O2	1.3231 (17)
C7—C8	1.4607 (17)	O2—H2	1.07 (2)
C8—O1	1.2354 (15)		
			
C2—C1—C5	117.35 (12)	C7—N1—C6	122.71 (11)
C2—C1—C6	120.89 (12)	C7—N1—H1	122.9 (9)
C5—C1—C6	121.76 (12)	C6—N1—H1	114.3 (9)
N2—C2—C1	123.74 (13)	C3—N2—C2	117.80 (12)
N2—C2—H2A	118.1	C14—C9—C10	119.28 (14)
C1—C2—H2A	118.1	C14—C9—C15	119.46 (13)
N2—C3—C4	122.74 (13)	C10—C9—C15	121.26 (13)
N2—C3—H3	118.6	C11—C10—C9	120.24 (15)
C4—C3—H3	118.6	C11—C10—H10	119.9
C3—C4—C5	118.83 (14)	C9—C10—H10	119.9
C3—C4—H4	120.6	C12—C11—C10	120.03 (16)
C5—C4—H4	120.6	C12—C11—H11	120
C4—C5—C1	119.53 (13)	C10—C11—H11	120
C4—C5—H5	120.2	C11—C12—C13	120.19 (15)
C1—C5—H5	120.2	C11—C12—H12	119.9
N1—C6—C1	111.52 (10)	C13—C12—H12	119.9
N1—C6—H6A	109.3	C12—C13—C14	120.03 (16)
C1—C6—H6A	109.3	C12—C13—H13	120
N1—C6—H6B	109.3	C14—C13—H13	120
C1—C6—H6B	109.3	C13—C14—C9	120.20 (16)
H6A—C6—H6B	108	C13—C14—H14	119.9
N1—C7—C7^i^	134.35 (7)	C9—C14—H14	119.9
N1—C7—C8	134.30 (12)	O3—C15—O2	123.58 (13)
C7^i^—C7—C8	91.35 (7)	O3—C15—C9	123.19 (14)
O1—C8—C7	135.25 (12)	O2—C15—C9	113.22 (12)
O1—C8—C8^i^	136.11 (7)	C15—O2—H2	111.9 (10)
C7—C8—C8^i^	88.64 (7)		
			
C5—C1—C2—N2	−0.52 (19)	C4—C3—N2—C2	−0.2 (2)
C6—C1—C2—N2	178.76 (11)	C1—C2—N2—C3	0.8 (2)
N2—C3—C4—C5	−0.7 (2)	C14—C9—C10—C11	0.3 (2)
C3—C4—C5—C1	0.9 (2)	C15—C9—C10—C11	−179.30 (13)
C2—C1—C5—C4	−0.35 (19)	C9—C10—C11—C12	−1.5 (2)
C6—C1—C5—C4	−179.62 (12)	C10—C11—C12—C13	1.1 (2)
C2—C1—C6—N1	98.57 (14)	C11—C12—C13—C14	0.5 (2)
C5—C1—C6—N1	−82.19 (14)	C12—C13—C14—C9	−1.6 (2)
N1—C7—C8—O1	−1.8 (3)	C10—C9—C14—C13	1.2 (2)
C7^i^—C7—C8—O1	178.55 (15)	C15—C9—C14—C13	−179.17 (13)
N1—C7—C8—C8^i^	178.45 (15)	C14—C9—C15—O3	−25.7 (2)
C7^i^—C7—C8—C8^i^	−1.20 (14)	C10—C9—C15—O3	153.96 (14)
C7^i^—C7—N1—C6	178.04 (17)	C14—C9—C15—O2	153.46 (12)
C8—C7—N1—C6	−1.5 (2)	C10—C9—C15—O2	−26.92 (18)
C1—C6—N1—C7	110.22 (13)		

Symmetry codes: (i) −*x*, *y*, −*z*+3/2.

### Hydrogen-bond geometry (Å, °)

**Table:**

*D*—H···*A*	*D*—H	H···*A*	*D*···*A*	*D*—H···*A*
N1—H1···O1^ii^	0.915 (16)	1.889 (17)	2.7697 (15)	161.0 (13)
O2—H2···N2	1.07 (2)	1.57 (2)	2.6380 (15)	178.0 (17)
C2—H2A···O3	0.95	2.68	3.3341 (18)	127

Symmetry codes: (ii) *x*, *y*−1, *z*.
